# Newborn RSV immunization rates and reasons compared to family COVID-19 and influenza immunization status

**DOI:** 10.1186/s12887-025-05889-x

**Published:** 2025-07-16

**Authors:** Joshua Somers, Brittany Hansen, Julia Burger, Stephen Aronoff, Brian Tuohy

**Affiliations:** Lewis Katz School of Medicine at Temple University, 3500 N Broad St, Philadelphia, PA 19140 USA

**Keywords:** Respiratory syncytial virus, Beyfortus, Parental decision-making, Vaccine hesitancy, Newborn immunization, Pediatric public health

## Abstract

**Background:**

Respiratory syncytial virus (RSV) is a common viral infection with the potential for severe illness in infants, leading to thousands of pediatric hospitalizations annually. In late 2023, Beyfortus (nirsevimab), a long-acting monoclonal antibody, became available to provide passive RSV immunization for all newborns meeting eligibility criteria. This study aimed to explore parental decision-making regarding RSV immunization, particularly in comparison to family uptake of COVID-19 and influenza vaccines, within an urban, predominantly Medicaid population in North Philadelphia.

**Methods:**

This qualitative study was conducted at Temple University Hospital and Temple Pediatric Care outpatient clinic. Semi-structured interviews were performed with 25 parents and primary caregivers of newborns who received RSV immunization during the 2023–2024 season. Participants were recruited from a retrospective list and interviewed by phone using a standardized questionnaire. Grounded Theory methodology was applied for data analysis using iterative coding to identify themes related to immunization perceptions and acceptance.

**Results:**

Participants expressed high levels of trust in healthcare providers and prioritized safety and efficacy when making immunization decisions. Parents accepted RSV immunization due to personal or family experiences with RSV, general desire to protect their newborns, or pediatrician recommendation. In contrast, COVID-19 and influenza vaccine decisions were less consistent. Concerns about side effects and perceived lack of effectiveness were common reasons for declining COVID-19 vaccines, despite similar motivations for protection. Social media and political beliefs had minimal reported influence. Parents reported a lack of consistent, reliable online sources for vaccine information, often relying on general internet searches. Many misunderstood immunization/vaccine efficacy, equating it with complete immunity rather than reduced disease severity.

**Conclusions:**

Parental acceptance of RSV immunization was driven by protective instincts for newborns and familiarity with RSV as a disease. Hesitancy toward COVID-19 vaccination stemmed largely from concerns about effectiveness and confusion around immunization/vaccine purpose. Trust in medical professionals influenced decision-making, though participants lacked a consistent source of immunization information. Improved public education on immunization efficacy and centralized access to trustworthy information may enhance immunization uptake and address ongoing hesitancy across all pediatric immunizations.

**Supplementary Information:**

The online version contains supplementary material available at 10.1186/s12887-025-05889-x.

## Introduction

With the release of Beyfortus (nirsevimab), a new respiratory syncytial virus (RSV) immunization available to families in late 2023, there has been unanticipated interest from patients in North Philadelphia. Prior to Beyfortus, only at-risk children received RSV protection with Synagis, a monthly injection. Beyfortus, is a single injection offered to any baby less than 8 months old and whose mother did not receive RSV vaccination in pregnancy. RSV is a viral infection that causes cough, congestion and often fever. 2–3% of RSV infected children will be hospitalized, possibly requiring oxygen, IV fluids, and mechanical ventilation. In total 58,000–80,000 pediatric patients are hospitalized each year due to RSV [1]. The highest risk group is children under 1 and those with chronic lung disease including prematurity. Beyfortus has been shown to decrease severe RSV infection by 75% [2]. The acceptance of Beyfortus has been most surprising in comparison to the family’s vaccination status of other seasonal viruses (COVID-19 and influenza).

Previous studies have explored trends in vaccine hesitancy and acceptance. Vaccine hesitancy varies significantly across demographic and political groups, with Black caregivers displaying higher hesitancy rates than White caregivers for pediatric influenza (42% vs. 21%) and COVID-19 (63% vs. 36%) [3]. Parents’ concerns largely center on vaccine safety, particularly long-term side effects, with 75.7% citing this as a reason for not vaccinating their child against COVID-19 [4]. Hesitancy was more prevalent among women, single parents, older individuals, those with lower incomes or education levels, and Republicans, who exhibited declining pro-vaccine attitudes during the pandemic [5, 6]. Conversely, Democrats and those who had received COVID-19 or influenza vaccines themselves were more likely to vaccinate their children [6]. Many parents expressed fatigue with contentious vaccine discourse on social media and preferred information from reputable health organizations [7]. Additionally, a notable portion of vaccine-hesitant parents (25%) preferred natural immunity through infection over vaccination [5]. There is limited literature when it comes to RSV immunization, but it has shown RSV immunization to be well received among parents of newborns [8, 9]. This study aims to better understand potential differences in acceptance of RSV immunization among newborns compared to the family’s vaccine status on COVID-19 and influenza vaccines while assessing some of the above factors and additional factors that may influence immunization/vaccination rates. Comparing RSV immunization with COVID-19 and influenza is valuable from a public health standpoint. Differences in acceptance may stem from variations in messaging as well as other factors, and understanding these can help improve immunization uptake and can help inform more targeted communication strategies and outreach efforts.

## Materials and methods

### Study design and setting

This qualitative study employed semi-structured interviews with parents and primary caregivers of newborns who received Beyfortus immunization for protection against RSV. This study adhered to the consolidated criteria for reporting qualitative research (COREQ) guidelines. This research is reported in accordance with the three COREQ domains: the research team and reflexivity, study design, and data analysis and findings. The study was conducted at Temple University Hospital (TUH), located in North Philadelphia, which serves an academic, urban, primarily Medicaid and uninsured population. Participants were retrospectively selected from two clinical settings: Temple Well Baby Nursery and Temple Pediatric Care outpatient clinic. The pediatric outpatient clinic serves approximately 14,000 patients aged 0–18, with 80% covered by Medicaid.

### Participant selection

Eligibility criteria included infants born at over 35 weeks of gestation who received the Beyfortus immunization during the 2023–2024 RSV season and were either born at or admitted to the Temple University Hospital System’s Well Baby Nursery or were a patient at Temple Pediatric Care. Exclusion criteria included refusal of Beyfortus immunization, age over eight months at the time of immunization, or discharge from Temple’s Infant Intensive Care Nursery (IICN). Newborns in the IICN were excluded because parental decision-making in this context may be influenced by the infants’ medical complexity, and consistent delivery of immunization counseling could not be ensured in this setting.

### Recruitment and data collection

A retrospective list of eligible participants was generated, and families were contacted via telephone. Verbal consent was obtained before conducting the interviews. All participants completed a standardized phone interview, approximately 15 min in length, to ensure consistency in data collection. Interviews were conducted over a four-month period and were recorded and transcribed for accuracy. Participants who completed the interview were entered into a raffle to win a gift card. A total of 25 interviews were conducted for the study—24 in English and one in Spanish, with the assistance of translation services. The interviews explored parental perceptions, experiences, and decision-making regarding Beyfortus immunization and family COVID-19 and influenza vaccination status. An English language version of the questionnaire is available as a supplementary file (Supplementary File 1).

### Data analysis

Grounded Theory methodology guided the data analysis process. An iterative coding process was used to identify themes and patterns from interview transcripts. Data were analyzed using constant comparative techniques to refine emerging themes based on response and concept saturation. Grounded theory was used in order to best explore people’s views on immunizations by identifying core categories of central themes of data. This strategy relies on theoretical saturation, which ensures that the developed theory is comprehensive and well supported by the data. As part of the coding process, each participant’s response to every question was first reviewed individually and summarized into key points. These summaries were then compared across participants’ responses to the same question to identify common patterns and ideas. Through several rounds of refinement, the key points were grouped into broader themes, which were used to develop a codebook. Throughout this iterative process, the emerging themes were regularly reviewed alongside the original responses to ensure they accurately reflected participants’ perspectives. This approach ultimately resulted in the core thematic categories used in the final analysis. Quantification was used to assess how strongly each central theme from the coding process was reflected in participants’ responses. Examining the frequency and consistency of each theme helped illustrate its relative strength and prominence across the dataset.

### Ethical considerations

The study was approved by the Institutional Review Board at Temple University (IRB No. 31445, approved on July 10, 2024). Verbal consent was obtained from all participants prior to data collection, and confidentiality of all responses was maintained throughout the study.

## Results

A total of 25 participants were interviewed as part of this study. The goal was to understand decision-making around the RSV immunization compared to other vaccines. Several key themes emerged, including the influence of personal and family experiences, the minimal impact of social media and politics, high trust in healthcare professionals, the lack of a consistent online source of information, and challenges in immunization messaging related to safety and efficacy.

### Impact of personal and family experiences

Participants who chose the RSV immunization primarily did so either to generally protect their newborn (36%) or due to personal and family experiences with RSV or a perceived elevated vulnerability of their newborn and thus protect their newborn (40%). One participant noted, “my middle child got RSV when he was younger and was in the hospital for 2 weeks - it was really bad. I didn’t want this to happen to my newborn so that’s why I got it.” Those with medical knowledge or awareness of health risks were more likely to desire immunization. Additionally, 16% of participants were influenced by their pediatrician’s recommendation, while 8% cited no specific reason. In contrast, family COVID-19 vaccination status was split. Participants who opted to vaccinate themselves or an older child did so out of a general desire for protection, without citing specific personal experiences. However, those who declined the COVID-19 vaccine often referenced concerns about side effects (18%) or skepticism about its effectiveness (64%), as they knew people who still got sick despite being vaccinated. For influenza, around 75% of participants chose to vaccinate themselves or their older children for general protection rather than due to personal experiences with the illness.

### Minimal influence of social media and politics

Only 12% of participants reported that social media or political factors influenced their vaccine decisions. Social media influence was mainly passive, with parents seeing posts about RSV, influenza, and COVID-19. Some mentioned that these posts reinforced their decision to vaccinate, but there was no strong indication that social media or political affiliation played a decisive role in their choices. This contrasts with earlier studies conducted during the COVID-19 pandemic, where political beliefs and social media had a more significant impact on vaccination decisions.

### Trust in healthcare professionals and sources of information

Participants overwhelmingly relied on healthcare professionals—including pediatricians, nurses, and pharmacists—for general vaccine information. The extent to which participants relied on medical professionals for information about each specific vaccine was not assessed. Trust in medical professionals was high, with 68% of participants expressing complete confidence in the information provided. However, 28% reported occasional doubts, though they still valued their provider’s guidance. Many participants noted that, while they trusted their doctors, they also sought additional information on their own. When searching for information on their own, there was no consistency in the external sources people used. Most participants conducted general internet searches and did not rely on specific websites, journals, or medical organizations for information. One participant stated, “I don’t use any specific websites, usually just browse the internet for hours through googling. I try to go to official websites, not wikipedia, for information from medical experts but it’s hard to find.” This lack of a go-to, trusted source highlights a gap in reliable, easily accessible vaccine information for the public.

### Challenges in immunization messaging

Participants identified safety and efficacy as the most important factors in their immunization decisions. Safety was understood primarily in terms of side effects, and 84% of participants reported never experiencing significant adverse effects from immunizations. The remaining participants noted mild reactions, such as injection site soreness, mild colds, or temporary discomfort. Efficacy, however, was more difficult for participants to define. Many equated efficacy with complete immunity, expecting that vaccines should entirely prevent infection. This misunderstanding contributed to skepticism, particularly with the COVID-19 vaccine, where breakthrough infections led some to believe the vaccine was ineffective. Among those whose newborn received RSV immunization but family did not vaccinate for COVID-19, 64% cited a perceived lack of effectiveness as their reason for declining the COVID-19 vaccine. This may suggest that immunization messaging needs to better explain how they primarily reduce the severity of illness rather than completely preventing infection. Clearer communication on what immunization efficacy truly means may help address misconceptions and improve vaccine confidence in the future.

## Discussion

This study aimed to explore differences in RSV immunization uptake compared to family COVID-19 and influenza vaccine uptake/status. One key finding was that personal experiences and exposures had a major impact on immunization decisions. If a parent, their child, or someone close to them had been affected by an illness or its vaccine, that experience strongly shaped their decision-making process. Personal experiences with RSV illness generally motivated participants to seek RSV immunization for their newborn, whereas experiences with the COVID-19 vaccine itself often discouraged family uptake of the COVID-19 vaccine. Parents were primarily concerned about the dangers of RSV infection and its potential impact on their newborns. In contrast, concerns about the COVID-19 vaccine centered on perceived effectiveness. Many participants had family or friends who still contracted COVID-19 despite being vaccinated, leading them to question its efficacy. For those families who chose to vaccinate against COVID-19, they noted reasons of general protection rather than due to personal experiences with the illness; this was the same reasoning used for influenza vaccination status of our participants.

It was notable that despite COVID-19’s widespread impact on individuals and families, it did not seem to drive vaccine uptake in the same way as RSV. This raises the question: Why is RSV different? One possible reason is that the RSV immunization, Beyfortus, is specifically for newborns. Parental instincts regarding newborn health may be fundamentally different from decisions made for older children, themselves or other adults. Studies have explored these parental motivations and found that when it comes to newborns specifically, parents have heightened responsibility and want to protect them as much as possible [10]. People are more empowered to take care of and protect their newborns and feel obligated to do whatever they can to protect them [11]. As a result, the combination of COVID-19 vaccine only offered to older population plus RSV illness having greater risk for the newborn population and RSV immunization now available, people are more likely to accept RSV immunization for their newborn. Another possible explanation for RSV immunization uptake is that people have had long-standing knowledge and personal experiences with RSV, which may have contributed to greater trust in the new immunization. Although both RSV immunization and the COVID-19 vaccine were novel when introduced to the parents, RSV was already a familiar illness. In contrast, COVID-19 was a novel virus when vaccine development was publicly announced, leaving people without prior experiences to rely on for decision-making. To summarize these ideas, both parental motivations to protect newborns and differences in familiarity among viruses may play a role in shaping trust in RSV immunization compared to the COVID-19 vaccine.

Another factor that may play a role in decision making regarding immunizations is where people get medical information from. Participants obtained immunization-related information from various sources, including healthcare professionals and online searches. Notably, social media and politics had little to no reported influence on their decisions. This contrasts with previous research conducted closer to the COVID-19 pandemic, where studies found that social media and political affiliation played a significant role in vaccine hesitancy [5, 6]. It is possible that social media influences people subconsciously, in that they may not recognize or acknowledge its impact on their decisions. Additionally, no participant in our study expressed disbelief in COVID-19 or framed immunization as a political issue. This suggests that opinions may have shifted since the height of the pandemic, when vaccine discourse was more politically charged.

When it came to trusted sources, healthcare professionals—including pediatricians, nurses, and pharmacists—played a role in decision-making. While there is documented mistrust in the healthcare system, particularly among disadvantaged groups, our findings indicated a higher level of trust in medical professionals than anticipated [12]. This could be attributed to the relationships built within Temple University Health System, where providers may be able to foster strong connections with patients. However, despite trust in medical professionals, participants still sought additional information independently. There was no consistency in external sources—most conducted Google searches and selected websites at random. No participant mentioned relying on a specific website, journal, or resource for vaccine information. This finding highlights two key issues: (1) many people trust the first source they find through a Google search, regardless of its credibility and (2) there is no widely recognized, centralized, and vetted resource for vaccine-related information. While people should have access to multiple sources, the absence of a go-to, reliable database for vaccine information suggests a need for a curated platform that directs individuals to reputable, evidence-based resources.

In addition to finding outside information about vaccines, participants have also developed their own views and thoughts on immunizations/vaccines. When evaluating vaccines, participants prioritized two factors: safety and efficacy. While their understanding of safety was accurate, there appeared to be a disconnect when it came to efficacy. Participants generally defined vaccine safety in terms of side effects. Because side effects are tangible and personally experienced, safety was an easier concept for them to assess. Most participants concluded a vaccine was “safe” if they did not experience major adverse reactions. Efficacy, however, was harder for participants to grasp. A common misconception was that a vaccine should completely prevent infection. This misunderstanding appears to stem from initial messaging during the COVID-19 pandemic, when many believed vaccination would provide full immunity. As a result, when vaccinated individuals still contracted COVID-19, people interpreted this as vaccine failure.

This misinterpretation persisted in our study. Among participants who chose to give their newborn RSV immunization but declined the COVID-19 vaccine for their family, 64% cited concerns about the COVID-19 vaccine’s effectiveness. This suggests an ongoing issue with how vaccine efficacy is communicated to the public. For example, when searching online, the phrase “RSV is a vaccine-preventable disease” can lead people to assume that vaccination completely eliminates the risk of infection. This can even be seen on both the U.S. Food and Drug Administration (FDA) and American Academy of Pediatrics (AAP) websites [13, 14]. In reality, vaccines are designed to reduce disease severity and spread, not necessarily to prevent all infections. One aspect that has been different surrounding RSV messaging, is that Beyfortus is not a traditional vaccine and because of this has been categorized as an “immunization.” This primarily comes from the mechanism of Beyfortus, being a monoclonal antibody providing passive immunization rather than activating the immune system (how vaccines work). It is possible this language was used both due to the mechanism of action and due to the public’s connotations with the word “vaccine.” This may also have had some influence on people’s receptiveness to RSV immunization compared to COVID-19 vaccine status. Although communication gaps may play a role, parental concerns may also be valid. Studies show that COVID-19 booster doses provide only limited added protection, as vaccine strains often do not match the currently circulating variants due to ongoing viral mutations [15]. Improving public understanding of what “immunization/vaccine efficacy” truly means is crucial in addressing some of the causes of vaccine hesitancy. Messaging strategies must evolve to ensure people grasp that vaccines mitigate risk rather than provide absolute protection.

Based on these findings, practical efforts should focus on improving general messaging around immunizations. There is a need for clearer communication about how different immunizations work and the type of protection they offer, which can enhance public understanding of immunization efficacy. The results also indicate greater acceptance of RSV immunization, suggesting it should be made widely accessible to parents. To support this, healthcare providers should have multiple opportunities to educate and encourage RSV immunization—before delivery, immediately after birth, and during the early postpartum months. Together, these strategies can help optimize vaccine uptake.

This study has some limitations. Individuals were only interviewed if they chose to immunize their newborns with the RSV immunization. As a result, this study did not assess the perspectives of those who declined the immunization, limiting our ability to compare decision-making and influences between those who accepted the RSV immunization and those who declined. This likely impacted the responses related to reasoning for immunizing, social media and political influence, and people’s trust among healthcare professionals. To ensure inclusivity regardless of language barriers, Temple University Language Line was used for interviews, however it is possible the translation over the phone was not perfect. With only 25 interviews completed, this cannot be completely generalized to the overall population, however it still provides insightful information.

## Conclusions

Overall, first-hand experiences or those of close friends and family had influence on vaccination/immunization choices. RSV immunization uptake was driven by some level of personal encounters with severe RSV cases, whereas hesitancy toward the COVID-19 vaccine stemmed from perceptions of its ineffectiveness based on breakthrough infections. People were more receptive to the RSV immunization potentially because RSV has been a known illness for a long time, whereas COVID-19’s novelty made its vaccine seem less trustworthy. Additionally, parental instincts likely played a role in RSV vaccine acceptance since it is given to newborns. Unlike previous studies, participants did not report being influenced by social media or political affiliation in their vaccine decisions. Instead, they relied primarily on healthcare professionals and general internet searches, though they lacked a consistent, reliable online resource for information. Lastly, while people correctly understood vaccine safety, many struggled to grasp efficacy, often expecting vaccines to completely prevent infection rather than reduce severity. When parents are clearly informed about the safety and benefits of RSV immunization, they are much more likely to accept it for their newborn. Trust in healthcare providers and effective communication about its role in preventing severe illness are key to promoting immunization uptake. The findings in this study suggest public health messaging around vaccines and immunization should explicitly address parental misconceptions about efficacy, clarifying that immunizations primarily prevent severe illness rather than all infections. Next steps based on this study can include a number of directions. Findings from this qualitative study were used to develop questions for a follow-up prospective study. Data collection for the 2024–2025 RSV season is currently in progress and includes participant demographics as well as detailed information on family COVID-19 and influenza vaccination status. Future studies should further explore factors influencing vaccine hesitancy, particularly focusing on parental motivations comparing newborns with other age children.

## Supplementary Information

Supplementary Material 1: English language version of the questionnaire used in the study.

## Data Availability

The datasets used and/or analysed during the current study are available from the corresponding author on reasonable request.

## Abbreviations

RSV: Respiratory Syncytial Virus
IICN: Infant Intensive Care Nursery
TUH: Temple University Hospital
FDA: U.S. Food and Drug Administration
AAP: American Academy of Pediatrics
