# Supplementary material for: Newborn RSV immunization rates and reasons compared to family COVID-19 and influenza immunization status
Source: BMC Pediatr. 2025 Jul 16;25:555. doi: 10.1186/s12887-025-05889-x (PMC12265380; doi:10.1186/s12887-025-05889-x)
Supplement: Supplementary file 1 — Supplementary Material 1: English language version of the questionnaire used in the study. [file 12887_2025_5889_MOESM1_ESM.docx]

**Questionnaire for Collecting Primary Data on Newborn RSV Immunization Rates and Reasons Compared to family COVID-19 and influenza Immunization Status**

**Instructions to Interviewer:**

- Introduce self, name and affiliation.
- Explain purpose and relevance of research.
- Ask participants if they have time and would like to answer some questions.
- Explain confidentiality and consent, obtain consent.

**Questions:**

1. Could you tell me a bit about your family and how many children you have?
2. Can you walk me through your thought process when deciding whether to vaccinate your child with the RSV immunization?
3. How does that compare to your decisions about influenza and COVID-19 vaccines?
4. What do you take into consideration and why? (regarding immunizations)
5. How has information from social media specifically influenced your decisions about vaccinations?
6. Can you provide an example of a memorable post or discussion that impacted your decision?
7. How do factors like your community, work environment, or educational background influence your decisions about vaccinations?
8. In what ways, if any, have political beliefs influenced your decision to vaccinate your children?
9. When we say “political beliefs”, what does that mean to you?
10. What does “public health” mean to you in this context with regards to vaccines?
11. How do you view your role and responsibility in protecting public health when deciding whether to vaccinate?
12. How do you decide which sources of health information are trustworthy?
13. Have you ever doubted the information provided by a healthcare professional? (please explain answer)
14. Could you describe a conversation with another parent or individual that significantly influenced your decision about vaccinations?
15. Are there cultural beliefs or practices that play a role in your decision-making process for vaccinations?
16. How have your previous experiences with vaccines, either positive or negative, shaped your current vaccination choices?
17. Given the shifting landscape of vaccine development and emerging diseases, how open are you to accepting new vaccines in the future? AND What would need to be true for you to feel comfortable with these decisions?
